# Prevalence and influence of hypouricemia on cardiovascular diseases in patients with rheumatoid arthritis

**DOI:** 10.1186/s40001-022-00888-5

**Published:** 2022-11-21

**Authors:** Yao-Wei Zou, Qian-Hua Li, Ying-Ying Zhu, Jie Pan, Jing-Wei Gao, Jian-Zi Lin, Tao Wu, Qian Zhang, Hu-Wei Zheng, Ying-Qian Mo, Jian-Da Ma, Lie Dai

**Affiliations:** 1 Department of Rheumatology, Sun Yat-sen Memorial Hospital, Sun Yat-sen University, 107 Yan Jiang West Road, Guangzhou, 510120 Guangdong People’s Republic of China; 2 Division of Clinical Research Design, Sun Yat-sen Memorial Hospital, Sun Yat-sen University, 107 Yan Jiang West Road, Guangzhou, 510120 Guangdong People’s Republic of China; 3 Department of Cardiology, Sun Yat-sen Memorial Hospital, Sun Yat-sen University, 107 Yan Jiang West Road, Guangzhou, 510120 Guangdong People’s Republic of China

**Keywords:** Cardiovascular disease, Rheumatoid arthritis, Serum uric acid, Hypouricemia, U-shaped curve

## Abstract

**Background:**

Serum uric acid (SUA) acts as an antioxidant and abnormally low SUA may raise the risk of developing atherosclerotic disorders. There is a U-shaped association between SUA with cardiovascular diseases (CVDs) in general population. However, the prevalence of hypouricemia and its influence on CVDs in rheumatoid arthritis (RA) remains unclear.

**Methods:**

This cross-sectional study collected clinical data from a Chinese RA cohort. Hypouricemia was defined as SUA ≤ 3.0 mg/dL, and hyperuricemia was defined as SUA ≥ 7.0 mg/dL. CVDs were defined as a history of angina pectoris, myocardial infarction, heart failure, stroke and peripheral arterial disease. Restricted cubic spline regression and logistic regression analysis were conducted to evaluate the associations between SUA levels and CVDs.

**Results:**

Among 1130 RA patients recruited, the mean age was 53.2 years and 79.0% were female. The prevalence of hypouricemia and hyperuricemia were 10.6% and 12.0%, respectively. RA patients with hyperuricemia had a higher rate of CVDs than normouricemic patients (27.9% vs. 7.1%, *P* < 0.05). Surprisingly, RA patients with hypouricemia also had a higher rate of CVDs (20.7% vs. 7.1%, *P* < 0.05) even without higher traditional cardiovascular risk factors. A U-shaped association between SUA levels and total CVDs was found (*P*_non-linear_ < 0.001). Multivariate logistic regression analysis revealed that compared with normouricemia, both hypouricemia [adjusted OR (AOR) = 4.707, 95% CI 2.570–8.620] and hyperuricemia (AOR = 3.707, 95% CI 2.174–6.321) were associated with higher risk of CVDs.

**Conclusions:**

Hypouricemia may be a potential risk factor of CVDs in RA patients

**Supplementary Information:**

The online version contains supplementary material available at 10.1186/s40001-022-00888-5.

## Background

Rheumatoid arthritis (RA) is a persistent, systemic, and immune-mediated inflammatory disease that increases mortality risk, shortens the life expectancy of 6 to 7 years, and causes 54% higher death rate [1]. Cardiovascular diseases (CVDs) are mostly to blame for the elevated risk of early mortality in RA patients. Previous researches have demonstrated that RA patients are up to two times more likely to developed CVDs than the general population [2], including 2.0-fold risk of myocardial infarction, 1.7-fold risk of congestive heart failure, and 2.0-fold risk of venous thrombotic disease [3, 4]. The European League Against Rheumatism (EULAR) suggests that RA patients should be estimated for CVD risk by using the risk score developed and validated in the general population [5]. But, only 50% of increased CVD risk in RA patients could be explained by the traditional cardiovascular risk factors [6]. If we used the prediction score algorithms developed in generals, which only included the traditional risk factors, to estimate the CVD risk in RA patients, it would definitely underestimate the CVD risk in RA [7]. By taking the independent effect of RA on CVD risk into account [2], the EULAR guideline suggests that the 10-year CVD risk estimates for RA patients should be 1.5-fold of the risk score calculated by the predictive model developed in the general population [5]. Even so, it has been claimed that using this multiplication factor did not reclassify as many patients as was anticipated into a more appropriate risk category [7, 8]. In order to precisely estimate CVD risk in RA, it is essential to identify new risk factors for CVDs in RA patients.

Serum uric acid (SUA) is the metabolic byproduct of purines generated by the breakdown of dietary or endogenous purines. The probable causative relationship between SUA and the risk of CVDs has been a topic of clinical and research interest in general population for past decades. Previous studies have demonstrated that hyperuricemia is linked to many health problems in generals, such as hypertension, obesity, dyslipidemia, type 2 diabetes mellitus (T2DM), chronic renal disease (CKD), and CVDs [9]. In 2018, the European Society of Cardiology/ European Society of Hypertension Guidelines for the management of arterial hypertension has considered SUA level as a CVD risk factor [10]. The measurement of SUA is also recommended as a part of the screening on hypertensive patients in 2021 European Society of Hypertension Guidelines [11]. Recently, due to the U-shaped association of SUA levels with the risk of CVDs and mortality found in general population, more attention has been paid to the effect of hypouricemia [12].

However, the effect of SUA has been less addressed in RA, probably due to the former believing that the coexistence of gout and RA is exceedingly rare [13]. Seldom studies reported that RA patients with hyperuricemia had a higher risk of CVDs and CVD mortality [14, 15]. There was no study to evaluate the relationship between hypouricemia and the risk of CVDs in RA patients. In this cross-sectional study, we firstly explored the prevalence of hypouricemia and its potential influence on CVD risk in RA patients.

## Methods

### Study design and participants

This cross-sectional study was conducted on our prospective RA cohort [16–20] at the Department of Rheumatology, Sun Yat-sen Memorial Hospital, China. Patients with a RA diagnosis of 2010 criteria [21] from June 2015 to March 2022 were recruited in this study. Patients with infections, malignancy, pregnancy, other autoimmune diseases, and gout (with or without urate-lowering treatment) were excluded. Ethical approval mandatory for this study was obtained from Ethics Committee at Sun Yat-sen Memorial Hospital (SYSEC-KY-KS-012, SYSEC-KY-KS-2020-208), along with informed consent from each patient.

### Data collection

Demographic and clinical information was gathered as we previously reported [16–20], including age, gender, active smoking, body mass index (BMI), disease duration, disease activity, physical function, radiographic indicators, comorbidities, and previous medications. Active RA was defined as the clinical disease activity index (CDAI) > 2.8. Physical function was assessed with the Stanford health assessment questionnaire disability index (HAQ-DI). Conventional radiographs of the bilateral hands and wrists were assessed with the Sharp/van der Heijde modified total Sharp score (mTSS).

Laboratory parameters of venous blood samples were measured using an autoanalyzer (Beckman AU5831 Biochemical Autoanalyzer, Beckman, USA). In humans, SUA 3.0–6.8 mg/dL is considered as normal range [22]. In this study, hypouricemia was defined as SUA level ≤ 3.0 mg/dL, while hyperuricemia was defined as SUA ≥ 7.0 mg/dL [23].

### CVD definition

The diagnosis of CVD was verified through a questionnaire survey and confirmation by medical record. CVDs were defined as a verified medical history of angina pectoris, myocardial infarction, heart failure, ischemic or hemorrhagic stroke, and peripheral arterial disease [24].

### Statistical analysis

Kruskal–Wallis *h* test and *χ*^2^ test were used to compare the differences in characteristics between three SUA categories. Bonferroni correction was further performed for multiple comparisons to correct the type I error. The connections between SUA levels and RA disease activity were examined using Pearson correlation analysis.

For the purpose to examine the non-linearity relationship between SUA levels and CVDs, restricted cubic spline (RCS) regression analysis was carried out in all RA patients and subgroups of gender. For limited cubic spline modeling, four knots (5th, 35th, 65th, and 95th percentiles of SUA levels) were utilized. Multivariate logistic regression analysis was conducted to evaluate the associations between SUA levels and CVDs. In model 2, age, gender (male or female), active smoking (yes or no), BMI, hypertension (yes or no), T2DM (yes or no), total cholesterol (TC), triglyceride (TG), high-density lipoprotein cholesterol (HDL-C), low-density lipoprotein cholesterol (LDL-C), serum albumin and CKD (yes or no) were adjusted for, while RA disease duration, rheumatoid factor (RF) positivity (yes or no), anti-cyclic citrullinated peptide antibody (ACPA) positivity (yes or no), erythrocyte sedimentation rate (ESR), C-reactive protein (CRP), CDAI, HAQ-DI, and mTSS were further added in model 3. Previous treatment, including treatment naïve (yes or no), glucocorticoid (yes or no), methotrexate (yes or no), leflunomide (yes or no), hydroxychloroquine (yes or no), sulfasalazine (yes or no), cyclosporine A (yes or no), tumor necrosis factor (TNF) inhibitors (yes or no), tocilizumab (yes or no), Janus kinase inhibitors (yes or no), statin (yes or no), and aspirin (yes or no) were added in model 4.

Sensitivity analysis was performed to reanalyze the relationship between SUA levels and CVDs according to different cut-off values of SUA including the results of RCS regression. Subgroup analysis was carried out according to age (< 60 or ≥ 60 years), gender (male or female), active smoking (yes or no), BMI (< 24 or ≥ 24 kg/m^2^), T2DM (yes or no), CKD (yes or no), TC (≤ 4.1, 4.1–5.2 or ≥ 5.2 mmol/L), TG (< 1.7, or ≥ 1.7 mmol/L), LDL-C (≤ 2.6, 2.6–3.4 or ≥ 3.4 mmol/L), HDL-C (< 1.0 or ≥ 1.0 mmol/L), treatment naïve (yes or no), and CRP (≤ 5, or > 5 mg/L). Potential interactions of these covariates with SUA levels were also tested. Statistical software packages SPSS and *R* were utilized for all analyses. Statistical significance was defined as a two-sided *P* value < 0.05.

## Results

### Demographic and clinical characteristics of RA patients

Among 1411 RA patients recruited in the cohort, 200 without complete laboratory measurement, 37 overlapped with other autoimmune diseases, 24 accompanied with malignancy, and 20 with gout were excluded. Totally, 1130 RA patients were eligible for analysis. Their mean age was 53.2 ± 12.6 years and 893 (79.0%) were female. The median disease duration was 62 months (range 20–121 months). There were 82.3% patients with active RA, and 22.9% treatment naïve patients who have not received glucocorticoid or disease-modifying anti-rheumatic drugs (DMARDs) therapy for 6 months before enrollment.

### Distribution of SUA in RA patients with different stratification

**Fig. 1:**
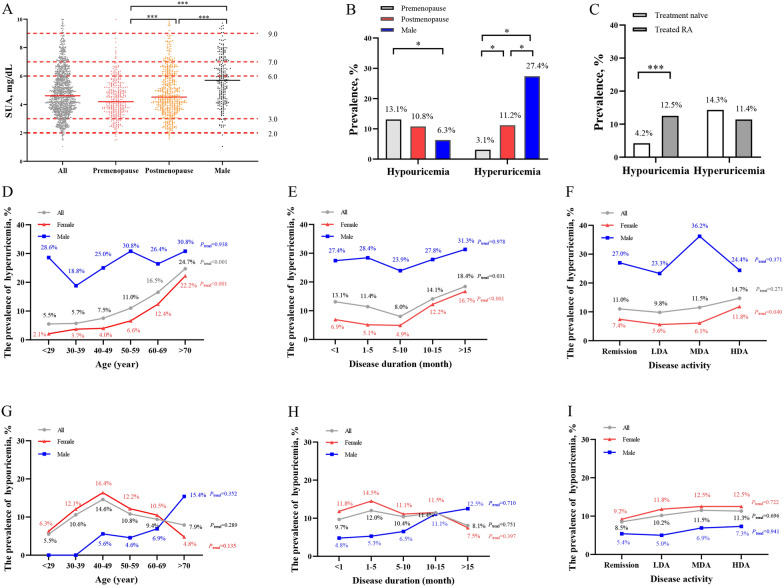
The levels of SUA and the prevalence of hypouricemia and hyperuricemia in RA patients with different stratification. The SUA levels in all, male, premenopausal and postmenopausal female RA patients **A**; The prevalence of hypouricemia and hyperuricemia in different gender **B** and treatment groups **C**; The prevalence of hyperuricemia in different age **D**, disease duration **E**, disease activity groups **F**; The prevalence of hypouricemia in different age **G**, disease duration **H**, disease activity groups **I**. *P < 0.05; **P < 0.01; ***P < 0.001. ^△^Treatment naïve, without previous corticosteroids or disease-modifying anti-rheumatic drugs treatment for 6 months before recruited. *RA* rheumatoid arthritis; Remission (CDAI ≤ 2.8), *LDA* low disease activity (2.8 < CDAI ≤ 10), *MDA* moderate disease activity (10 < CDAI ≤ 22), *HAD* high disease activity (CDAI > 22)

The mean SUA level in all RA patients was 4.92 ± 1.72 mg/dL. The SUA level in males was significantly higher than that in females (5.79 ± 1.85 mg/dL vs. 4.68 ± 1.60 mg/dL, *P* < 0.001). The SUA level in postmenopausal female RA patients was higher than that in premenopausal patients (4.88 ± 1.74 mg/dL vs. 4.39 ± 1.34 mg/dL, *P* < 0.001, Fig. 1A).

The prevalence of hyperuricemia in RA patients was 12.0% (136/1130). The male RA patients had the highest prevalence of hyperuricemia than both females after and before menopause (27.4% vs. 11.2% vs. 3.1%, *P* < 0.05, Fig. 1B). The prevalence of hyperuricemia increased with age, disease duration, and disease activity in female RA patients, but not in male patients (Fig. 1D–F). The prevalence of hyperuricemia showed no difference between treatment naïve and treated RA patients (Fig. 1C).

On the other hand, the prevalence of hypouricemia in RA patients was 10.6% (120/1130). The premenopausal female RA patients had a higher prevalence of hypouricemia than males (13.1% vs. 6.3%, *P* < 0.05, Fig. 1B). The treated RA patients had a higher prevalence of hypouricemia than those treatment naïve patients (12.5% vs. 4.2%, *P* < 0.001, Fig. 1C). There was no significant difference in hypouricemia prevalence among different age, disease duration, or disease activity groups (Fig. 1G–I).

**Fig. 2:**
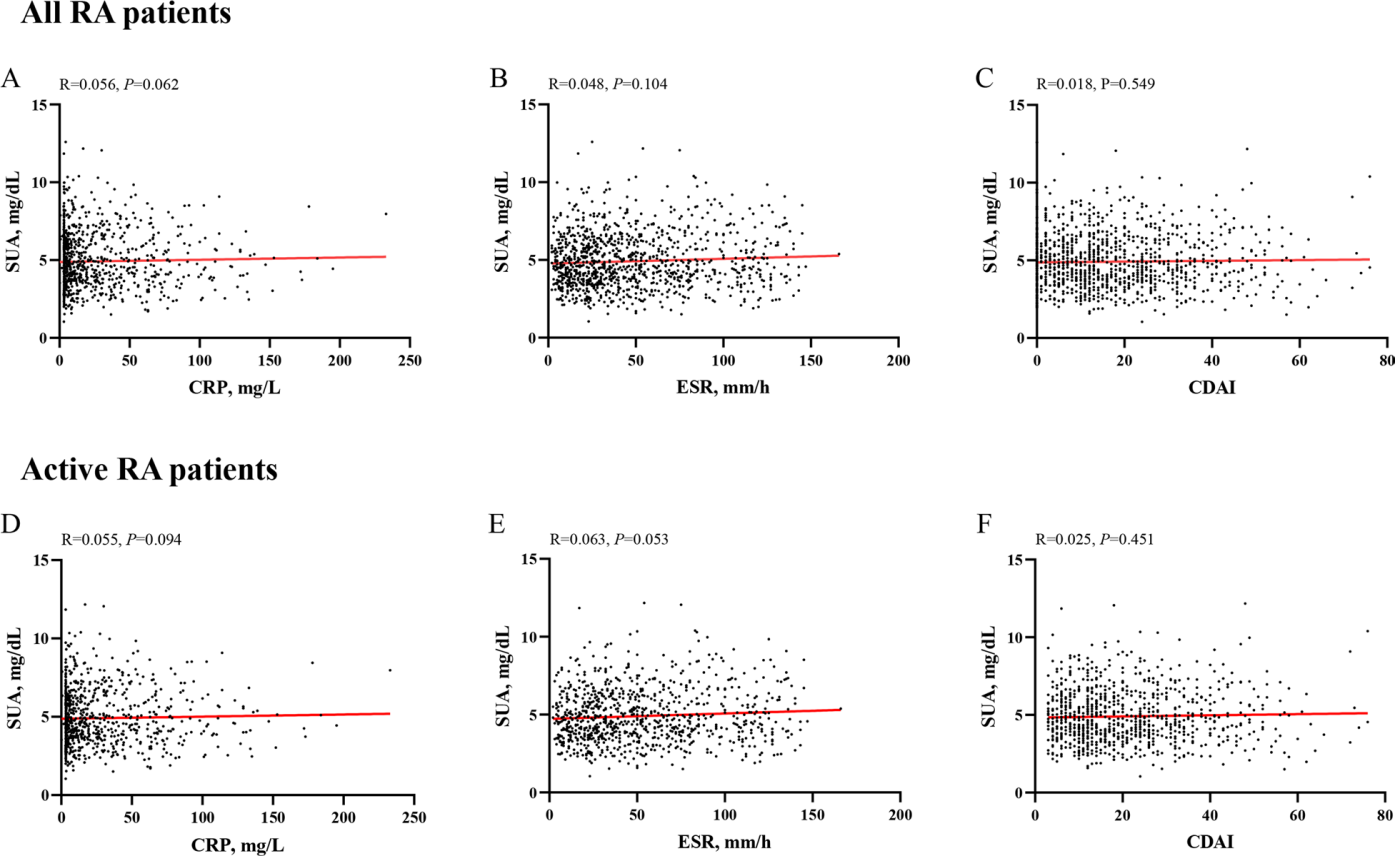
The associations between SUA levels and RA disease activity. The associations between SUA levels and CRP **A**, ESR **B**, CDAI **C** in all RA patients. The associations between SUA levels and CRP **D**, ESR **E**, CDAI **F** in active RA patients. *SUA* serum uric acid, *CRP* C-reactive protein, *ESR* erythrocyte sedimentation rate, *CDAI* clinical disease activity index

Pearson correlation analysis showed no significant association between SUA levels and RA disease activity in all RA or active RA patients (Fig. 2).

### Clinical characteristics of RA patients among different SUA groups

Compared with normouricemic group, RA patients with hyperuricemia were older (mean 58.3 years vs. 52.5 years), more male (47.8% vs. 18.0%), and having higher levels of ESR (median 49 mm/h vs. 35 mm/h) and CRP (median 12.85 mg/L vs. 5.41 mg/L), and higher HAQ-DI (median 0.75 vs. 0.38, all *P* < 0.05, Table 1). But, RA patients with hypouricemia showed no difference of demographic and RA disease characteristics, except for lower proportion of treatment naïve (9.2% vs. 24.1%) and higher proportion of previous treatment of glucocorticoid (60.8% vs. 46.9%), methotrexate (74.2% vs. 55.7%), and leflunomide (75.8% vs. 40.5%, all *P* < 0.05).

**Table 1:** Comparisons of demographic and clinical characteristics among RA patients with different SUA levels

Characteristics	All RA patients (*n* = 1130)	Hypouricemia (*n* = 120)	Normouricemia (*n* = 874)	Hyperuricemia (*n* = 136)	*P*
Female, *n* (%)	893 (79.0)	105 (87.5)	717 (82.0)	71 (52.2)^ab^	< 0.001
Age, years	53.2 ± 12.6	52.7 ± 11.5	52.5 ± 12.6	58.3 ± 12.0^a^	< 0.001
Disease duration, months	62 (20,121)	57 (21,120)	62 (22,120)	74 (15,141)	0.736
Positive RF, *n* (%)	794 (70.3)	82 (68.3)	611 (69.9)	101 (74.3)	0.520
Positive ACPA, *n* (%)	819 (72.5)	87 (72.5)	634 (72.5)	98 (72.1)	0.993
ESR, mm/h	37 (20,70)	43 (23,68)	35 (18,67)	49 (25,85)^a^	0.001
CRP, mg/L	6.44 (3.23,28.50)	7.75 (3.23,36.45)	5.41 (3.16,25.03)	12.85 (3.30,42.90)^a^	0.001
CDAI	14 (5 26)	14 (6 27)	13 (5 25)	17 (7 28)	0.091
Active RA, *n* (%)	930 (82.3)	103 (85.8)	713 (81.6)	114 (83.8)	0.459
HAQ-DI	0.38 (0.00,1.13)	0.44 (0.00,1.34)	0.38 (0.00,1.00)	0.75 (0.13,1.63)^a^	0.001
mTSS	11 (4.34)	10 (3.33)	11 (4.33)	15 (3.45)	0.332
Previous medications					
Treatment naïve^△^, *n* (%)	259 (22.9)	11 (9.2)^a^	211 (24.1)	37 (27.2)^b^	0.001
Glucocorticoid, *n* (%)	549 (48.6)	73 (60.8)^a^	410 (46.9)	66 (48.5)	0.017
Methotrexate, *n* (%)	642 (56.8)	89 (74.2)^a^	487 (55.7)	66 (48.5)^b^	< 0.001
Leflunomide, *n* (%)	492 (43.5)	91 (75.8)^a^	354 (40.5)	47 (34.6)^b^	< 0.001
Hydroxychloroquine, *n* (%)	227 (20.1)	20 (16.7)	188 (21.5)	19 (14.0)	0.076
Sulfasalazine, *n* (%)	59 (5.2)	4 (3.3)	48 (5.5)	7 (5.1)	0.608
Cyclosporine A, *n* (%)	40 (3.5)	1 (0.8)	35 (4.0)	4 (2.9)	0.195
TNF inhibitors, *n* (%)	29 (2.6)	7 (5.8)	19 (2.2)	3 (2.2)	0.057
Tocilizumab, *n* (%)	34 (3.0)	4 (3.3)	27 (3.1)	3 (2.2)	0.834
Janus kinase inhibitors, *n* (%)	36 (3.2)	3 (2.5)	29 (3.3)	4 (2.9)	0.879
Statin, *n* (%)	95 (8.4)	7 (5.8)	75 (8.6)	13 (9.6)	0.522
Aspirin, *n* (%)	33 (2.9)	3 (2.5)	25 (2.9)	5 (3.7)	0.835
CVD risk factors					
Active smoking, *n* (%)	190 (16.8)	11 (9.2)	125 (14.3)	54 (39.7)^ab^	< 0.001
BMI, kg/m^2^	21.9 ± 3.0	21.1 ± 2.7^a^	21.9 ± 3.0	22.4 ± 3.2^b^	0.004
Hypertension, *n* (%)	363 (32.1)	39 (32.5)	264 (30.2)	60 (44.1)^a^	0.005
T2DM, *n* (%)	158 (14.0)	18 (15.0)	102 (11.7)	38 (27.9)^ab^	< 0.001
TC, mmol/L	5.04 ± 1.19	5.11 ± 1.05	5.02 ± 1.21	5.12 ± 1.20	0.546
TG, mmol/L	1.19 ± 0.79	0.98 ± 0.44^a^	1.16 ± 0.76	1.58 ± 1.09^ab^	< 0.001
LDL-C, mmol/L	3.15 ± 0.85	3.11 ± 0.75	3.13 ± 0.86	3.32 ± 0.87^a^	0.046
HDL-C, mmol/L	1.37 ± 0.39	1.50 ± 0.41^a^	1.39 ± 0.39	1.19 ± 0.33^ab^	< 0.001
CKD, *n* (%)	33 (2.9)	0 (0)	14 (1.6)	19 (14.0)^ab^	< 0.001
Serum albumin, g/L	34.3 ± 4.8	33.3 ± 5.2^a^	34.8 ± 4.8	33.3 ± 4.6^a^	0.001
SUA, mg/dL	4.91 ± 1.72	2.48 ± 0.36^a^	4.74 ± 1.05	8.18 ± 1.12^ab^	< 0.001

^△^Treatment naïve, without previous corticosteroids or disease-modifying anti-rheumatic drugs treatment for 6 months before recruited; active RA was defined as CDAI > 2.8

*RF* rheumatoid factor, *ACPA* anti-cyclic citrullinated peptide antibody, *ESR* erythrocyte sedimentation rate, *CRP* C-reactive protein, *CDAI* clinical disease activity index, *HAQ-DI* health assessment questionnaire disability index, *mTSS* modified total Sharp score, *TNF* tumor necrosis factor, *CVD* cardiovascular disease, *BMI* body mass index, *T2DM* type 2 diabetes mellitus, *TC* total cholesterol, *TG* triglyceride, *HDL-C* high-density lipoprotein cholesterol, *LDL-C* low-density lipoprotein cholesterol, *CKD* chronic kidney disease, *SUA* serum uric acid

^a^Compared with normouricemic group

^b^Compared with hypouricemic group

As expected, compared with normouricemic group, RA patients with hyperuricemia had more traditional cardiovascular risk factors, including higher proportion of active smoking (39.7% vs. 14.3%), hypertension (44.1% vs.30.2%), T2DM (27.9% vs. 11.7%), and CKD (14.0% vs. 1.6%), and higher level of LDL-C (mean 3.32 mmol/L vs. 3.13 mmol/L) and TG (mean 1.58 mmol/L vs. 1.16 mmol/L, all *P* < 0.05). However, RA patients with hypouricemia showed higher levels of HDL-C (mean 1.50 mmol/L *vs.* 1.39 mmol/L), lower levels of TG (mean 0.98 mmol/L vs. 1.16 mmol/L), BMI (mean 21.1 kg/m^2^ vs. 21.9 kg/m^2^), and serum albumin (mean 33.3 g/L *vs.* 34.8 g/L, all *P* < 0.05).

### CVD in RA patients among different SUA groups

**Fig. 3:**
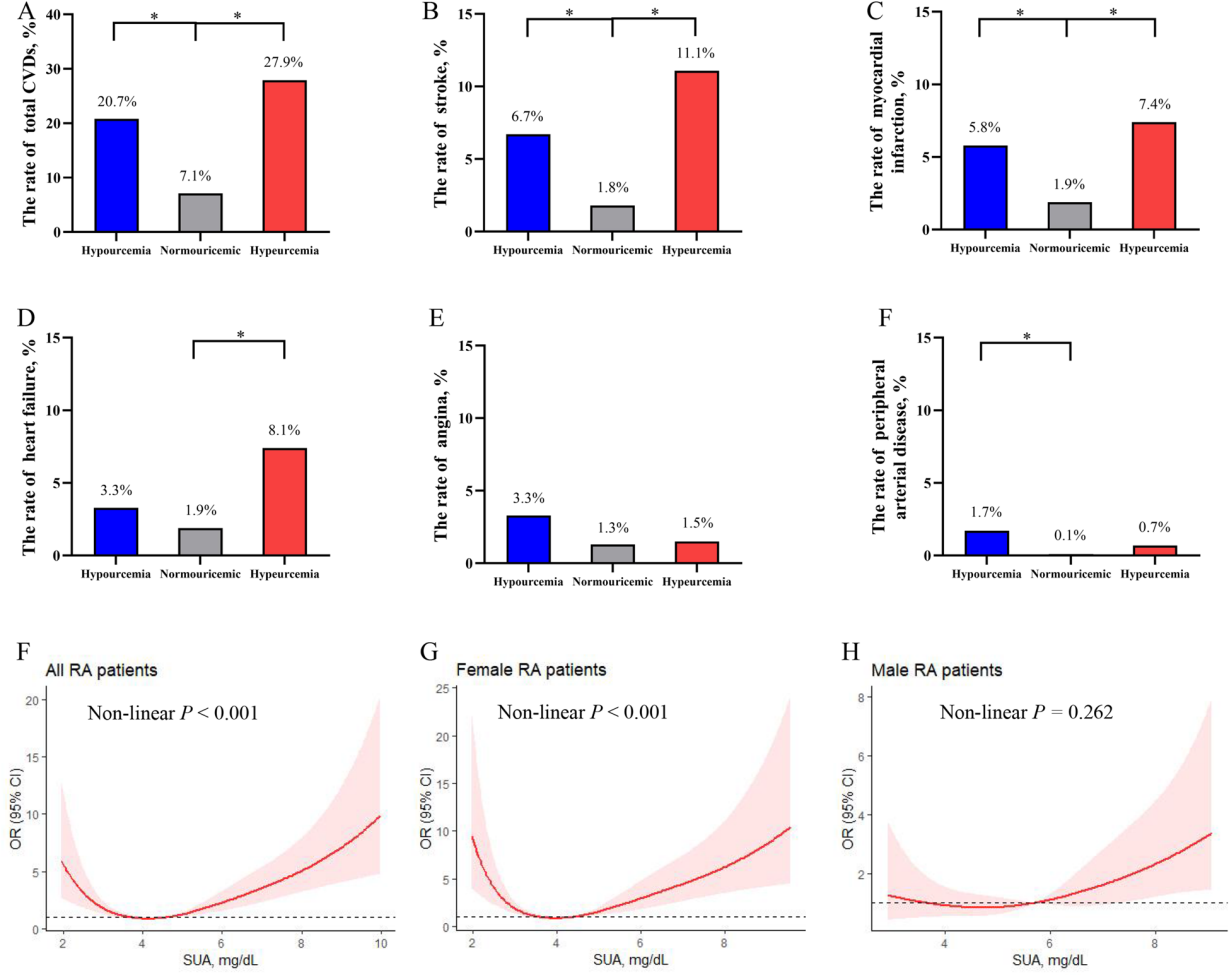
The associations between SUA levels and CVDs in RA patients. The rates of total CVDs **A**, stroke **B**, myocardial infarction **C**, heart failure **D**, angina **E,** and peripheral arterial disease **F** in RA patients with different SUA levels; the restricted cubic spline regression analysis shows the relationship between SUA levels and CVDs in all **F**, female **G,** and male RA patients **H**; OR (95% CI) was estimated with univariate logistic regression analysis. The red line represents the OR and the pink shaded area is the 95% CI. *P < 0.05; **P < 0.01; ***P < 0.001. *CVD* cardiovascular disease, *SUA* serum uric acid, *RA* rheumatoid arthritis, *OR* odds ratio, *CI* confidence interval

There were 125 (11.1%) patients concomitated with CVDs, including stroke (3.5%), myocardial infarction (3.0%), heart failure (2.7%), angina pectoris (1.5%), and peripheral arterial disease (0.4%). As expected, compared with normouricemic group, RA patients with hyperuricemia had a higher rate of total CVDs (27.9% vs. 7.1%), including stroke (11.1% vs. 1.8%), myocardial infarction (7.4% vs. 1.9%), and heart failure (8.1% vs. 1.9%, all *P* < 0.05, Fig. 3). Surprisingly, RA patients with hypouricemia also had a higher rate of total CVDs (20.7% vs. 7.1%), including stroke (6.7% vs. 1.8%), myocardial infarction (5.8% *vs.* 1.9%), and peripheral arterial disease (1.7% vs. 0.1%, all *P* < 0.05).

In turn, compared with those without CVDs, RA patients with CVDs had higher levels of SUA (mean 5.57 mg/dL vs. 4.83 mg/dL), higher rate of hypouricemia (20.0% vs. 9.5%), and hyperuricemia (30.4% vs. 9.8%). In addition, RA patients with CVDs were older (mean 63.1 years vs. 52.0 years), more male (30.4% vs. 19.8%), and having more serious RA disease and more traditional cardiovascular risk factors (all *P* < 0.05, Additional file 1: Table S1).

### Associations between SUA levels and CVD in RA patients

**Fig. 4:**
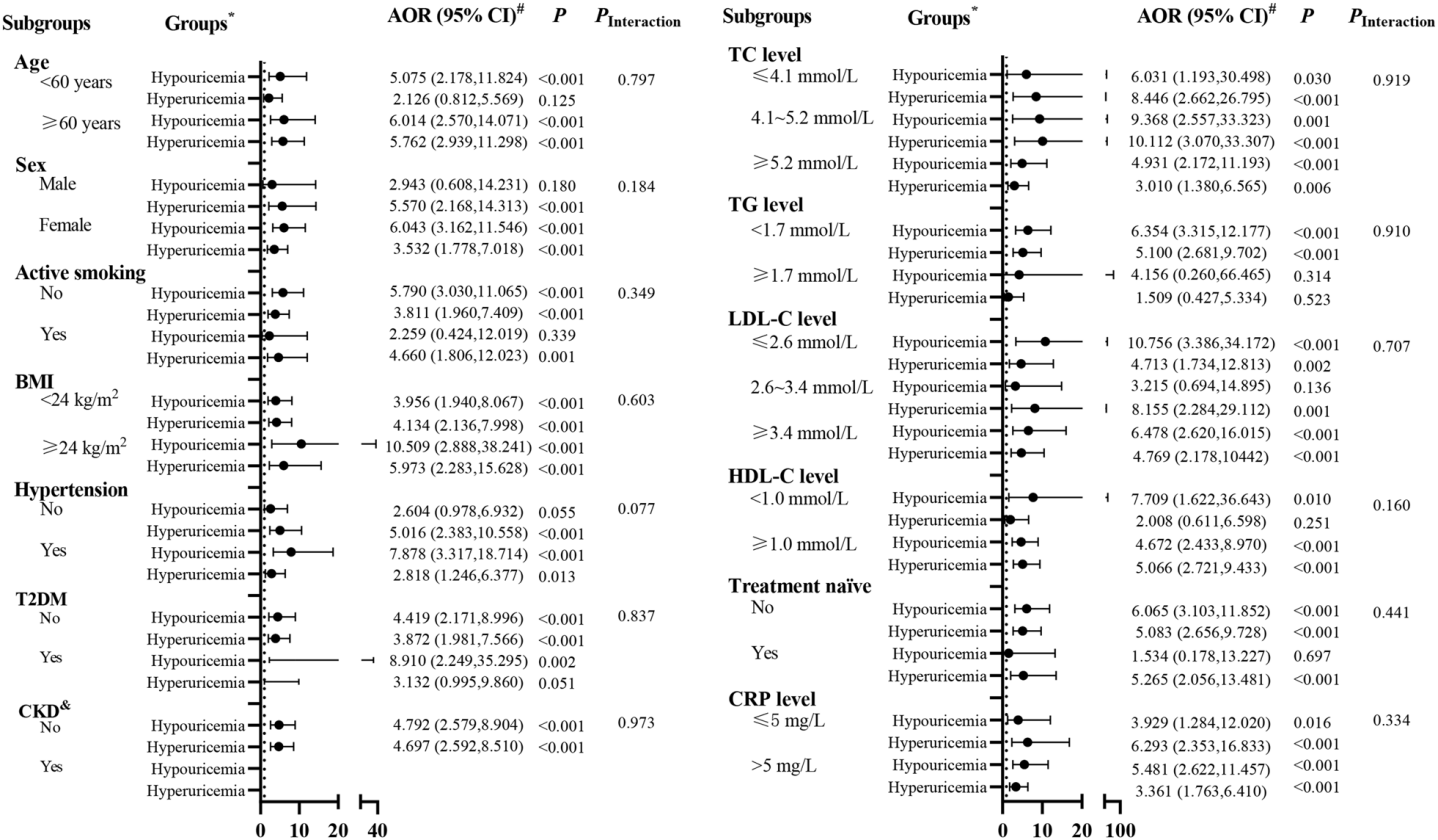
Subgroup analyses of the associations of SUA levels with CVDs in RA patients. *BMI* body mass index, *T2DM*, type 2 diabetes mellitus, *CKD* chronic kidney disease, *TC* total cholesterol, *TG* triglyceride, *HDL-C* high-density lipoprotein cholesterol, *LDL-C* low-density lipoprotein cholesterol, *TNF* tumor necrosis factor, *AOR* adjusted OR, *CI* confidence interval. *Normouricemic group was used as the reference in each subgroup analysis. #Adjusted by age, gender (male or female), active smoking (yes or no), BMI, hypertension (yes or no), T2DM (yes or no), TC, TG, LDL-C, HDL-C, serum albumin and CKD (yes or no), disease duration, RF positivity (yes or no), ACPA positivity (yes or no), ESR, CRP, CDAI, HAQ-DI, mTSS and previous treatment, including treatment naïve (yes or no), glucocorticoid (yes or no), methotrexate (yes or no), leflunomide (yes or no), hydroxychloroquine (yes or no), sulfasalazine (yes or no), cyclosporine A (yes or no), TNF inhibitors (yes or no), tocilizumab (yes or no), Janus kinase inhibitors (yes or no), statin (yes or no), aspirin (yes or no). & There was no patient with hypouricemia in CKD group

The results of RCS regression showed a U-shaped relationship between SUA levels and total CVDs in all RA patients and female RA patients (both non-linear *P* < 0.001, Fig. 3F, G), but not in male RA patients (non-linear *P* = 0.262, Fig. 3H). After fully adjusted for the potential covariates (model 4, Table 2), multivariate logistic regression analysis revealed that compared with normouricemia, both hypouricemia [adjusted OR (AOR) = 4.707, 95% CI 2.570–8.620] and hyperuricemia (AOR = 3.707, 95% CI 2.174–6.321) were associated with higher CVDs in RA patients (both *P* < 0.05). The result of sensitivity analyses was consistent among different cut-off values (Additional file 1: Table S2). Among subgroup analyses, the association of hypouricemia and hyperuricemia with CVDs prevalence remained almost similar to all RA patients (Fig. 4).

**Table 2:** Multivariate logistic regression analysis of the association between SUA levels and CVD in RA patients

Characteristics	Model 1		Model 2		Model 3		Model 4	
	OR (95% CI)	*P*	AOR (95% CI)	*P*	AOR (95% CI)	*P*	AOR (95% CI)	*P*
Hypouricemia	3.447 (2.068,5.743)	< 0.001	4.015 (2.262,7.127)	< 0.001	4.174 (2.350,7.414)	< 0.001	4.707 (2.570,8.620)	< 0.001
Normouricemia	Ref		Ref		Ref		Ref	
Hyperuricemia	5.078 (3.222,8.004)	< 0.001	3.133 (1.896,5.179)	< 0.001	3.198 (1.931,5.295)	< 0.001	3.707 (2.174,6.321)	< 0.001

Model 1: Unadjusted; Model 2: Adjusted for age, gender (male or female), active smoking (yes or no), BMI, hypertension (yes or no), T2DM (yes or no), TC, TG, LDL-C, HDL-C, serum albumin and CKD (yes or no); Model 3: Adjusted for model 2 covariates plus RA disease duration, RF positivity (yes or no), ACPA positivity (yes or no), ESR, CRP, CDAI, HAQ-DI, mTSS; Model 4: Adjusted for model 3 covariates plus previous treatment, including treatment naïve (yes or no), glucocorticoid (yes or no), methotrexate (yes or no), leflunomide (yes or no), hydroxychloroquine (yes or no), sulfasalazine (yes or no), cyclosporine A (yes or no), TNF inhibitors (yes or no), tocilizumab (yes or no), Janus kinase inhibitors (yes or no), statin (yes or no), aspirin (yes or no)

*CVD* cardiovascular disease, *SUA* serum uric acid, *RF* rheumatoid factor, *ACPA* anti-cyclic citrullinated peptide antibody, *ESR* erythrocyte sedimentation rate, *CRP, C*-reactive protein, *CDAI* clinical disease activity index, *HAQ-DI* health assessment questionnaire disability index, *mTSS* modified total Sharp score, *TNF* tumor necrosis factor *T2DM* type 2 diabetes mellitus, *TC* total cholesterol, *TG* triglyceride, *HDL-C* high-density lipoprotein cholesterol, *LDL-C* low-density lipoprotein cholesterol; *CKD* chronic kidney disease, *OR* odds ratio, *AOR* adjusted OR; *CI* confidence interval

## Discussion

In this cross-sectional investigation, we firstly reported that 10.6% RA patients had hypouricemia. RA patients with hypouricemia had 4.7-fold higher prevalence of CVDs than those with normouricemia. These findings highlight that hypouricemia may be a new potential risk factor of CVDs in RA patients.

Hyperuricemia and gout have grown to be a significant global public health problem. A number of researches have investigated the link between hyperuricemia and harmful outcomes in general population. Several studies demonstrated a positive association between hyperuricemia with all-cause and CVD mortality [25, 26], as well as higher risk of CVDs in general [12]. There are a number of potential explanations for the link between high SUA levels and elevated CVD hazard, including the coexistence of traditional cardiovascular risk factors and the direct interaction of uric acid with several metabolic processes linked to CVDs [27]. There were seldom studies addressing hyperuricemia in RA. Research conducted in US veterans (90% male) reported that 17.0% RA patients had hyperuricemia [14], which was approached the prevalence rate of hyperuricemia in the general men in US (20.0%) [28]. RA patients with hyperuricemia possessed a higher comorbidity burden and hyperuricemia is positively correlated with CVDs and CVD mortality [14, 15]. In our study, there were 79.0% females in 1130 RA patients. The prevalence of hyperuricemia in all, female, and male RA patients were 12.0%, 8.0%, and 27.4%, respectively, while 14.0% in Chinese adults [29]. RA patients with hyperuricemia had more traditional cardiovascular risk factors together with 3.7-fold higher prevalence of CVDs than normouricemic patients.

Recent studies reported U-shaped connections between SUA levels and CVDs, as well as mortality in general population [12]. The harmful effects of hypouricemia are being attracted more and more attention. SUA acts as an antioxidant by accelerating the conversion of superoxide to hydrogen peroxide, reducing the amount of available superoxide, and preventing the dangerous interaction of superoxide with nitric oxide. Therefore, low SUA may raise the risk of developing atherosclerotic disorders due to a diminished antioxidant capacity [30]. A previous study reported that there were 1.2% subjects with low SUA (2–3 mg/dL) in Taiwan elderly (≥ 65 years) and those with low SUA had 20% and 35% higher risks of all-cause and CVD mortality, respectively [31]. Another study conducted in Korean adults showed that there were 1.4% males and 6.3% females with low SUA (< 3.5/2.5 mg/dL for male/female) and those with low SUA also had higher risk of mortality [32]. All these studies indicated that abnormally low SUA level can attribute to potentially harmful outcomes. However, there was no study on hypouricemia in RA patients. In this study, we firstly reported the rate of hypouricemia in all, female, and male RA patients were 10.6%, 11.8%, and 6.3%, respectively. RA patients with hypouricemia showed 4.7-fold higher prevalence of CVDs than normouricemic patients even without higher traditional cardiovascular risk factors. SUA levels showed a U-shaped association with CVDs in RA patients.

Inflammation is the central part of immunological pathophysiology in RA which is also critical in the development of CVD [24]. Chronic inflammatory response in RA is a very energy-intensive process that causes a hypermetabolic and catabolic state with higher resting energy consumption, resulting in weight loss, poor nutrition, and even cachexia [33]. Low SUA level may indicate those who are undernourished [34]. A recent study showed that low SUA is, itself, not adverse to health and may instead reflect other adverse biologic processes (such as weight loss or sarcopenia) that are associated with mortality in general population [35]. In our study, RA patients with hypouricemia had lower BMI and serum albumin, which implied poor nutrition. In addition, some DMARDs for RA, such as leflunomide, may reduce SUA concentration [36]. Our study showed a higher prevalence of hypouricemia in treated RA patients than that in treatment naïve patients (12.5% vs. 4.2%). However, multivariate logistic regression revealed that hypouricemia is still associated with higher CVDs after adjusted these confounders, including active RA, BMI, serum albumin, and medication. All these findings suggested that hypouricemia may be a potential associated factor for CVDs in RA patients.

RA-related systemic inflammation plays important roles in determining cardiovascular risk and a complex relationship between LDL-C and cardiovascular risk [37]. Previous studies demonstrated that low LDL-C levels were connected to increased CVD risk in RA patients, and this connection was dubbed the "lipid paradox" [38]. Differences in the association between lipid levels with CVD risk in RA might be linked to inflammation [39]. As disease activity increases, inflammation (such as CRP) increases and lipid levels (such as LDL-C) decrease, and anti-rheumatic medication dampens inflammation, which is accompanied by an inverse increase in lipid levels [24]. Thus, a reduction in LDL-C levels due to active disease does not imply a decrease of cardiovascular risk. In our study, the levels of SUA showed no association with RA disease activity. Other clinical investigations also demonstrated similar results [14, 40]. These results indicated that hypouricemia, different from low LDL-C, may be an independent risk factor of CVDs in RA patients, and further prospective cohort study is worth in future.

This study had several limitations. First, given the nature of cross-sectional investigation, the causality between SUA levels and CVD outcome cannot be determined even we have carefully controlled possible risk variables. Second, food consumption may affect the SUA level in which 20% of human SUA pool come from exogenous food consumption especially alcohol, purine-rich meat, and sugar-sweetened beverages [41, 42]. Our study did not investigate food consumption, which might have an impact on SUA levels due to a lack of pertinent data. Third, unlike the general population where the U-shaped associations between SUA levels and CVDs are shown in both males and females [12], our study showed the U-shaped correlation only in female RA patients. The sample size of male RA patients was less (*n* = 237) which may be not large enough to find a significant difference. A future large-scale multicenter prospective investigation is required to address these limitations.

## Conclusions

In summary, this investigation reports the prevalence of hypouricemia in RA patients. RA patients with hypouricemia have 4.7-fold higher prevalence of CVDs than those with normouricemia. Hypouricemia may be a new potential risk factor of CVDs in RA patients. Further prospective cohort study is worth in future.

## Supplementary Information

**Additional file 1: Table S1.** Comparisons of clinical characteristics between RA patients with and without CVD. **Table S2.** Logistic regression analysis of the associations between SUA levels and CVD in RA patients.

## Data Availability

The datasets used and/or analyzed during the current study are available from the corresponding author Prof. Dai on reasonable request.

## Abbreviations

RA: Rheumatoid arthritis
CVD: Cardiovascular disease
EULAR: European League Against Rheumatism
SUA: Serum uric acid
T2DM: Type 2 diabetes mellitus
CKD: Chronic kidney disease
BMI: Body mass index
CDAI: Clinical disease activity index
HAQ-DI: Health assessment questionnaire disability index
mTSS: Modified total Sharp score
RCS: Restricted cubic spline
TC: Total cholesterol
TG: Triglyceride
HDL-C: High-density lipoprotein cholesterol
LDL-C: Low-density lipoprotein cholesterol
RF: Rheumatoid factor
ACPA: Anti-cyclic citrullinated peptide antibody
ESR: Erythrocyte sedimentation rate
CRP: C-reactive protein
OR: Odds ratio
AOR: Adjusted OR
