# Supplementary material for: Prevalence and influence of hypouricemia on cardiovascular diseases in patients with rheumatoid arthritis
Source: Eur J Med Res. 2022 Nov 21;27:260. doi: 10.1186/s40001-022-00888-5 (PMC9677667; doi:10.1186/s40001-022-00888-5)
Supplement: Supplementary file 1 — Additional file 1: Table S1. Comparisons of clinical characteristics between RA patients with and without CVD. Table S2. Logistic regression analysis of the associations between SUA levels and CVD in RA patients. [file 40001_2022_888_MOESM1_ESM.docx]

**Additionl file materials**

**Additionl file 1: Table S1 Comparisons of clinical characteristics between RA patients with and without CVD**

| Characteristics | RA without CVD  (n =1005) | RA with CVD  (n =125) | *P* |
| --- | --- | --- | --- |
| Female, n (%) | 806 (80.2) | 87 (69.6) | 0.006 |
| Age, years | 52.0±12.4 | 63.1±8.7 | <0.001 |
| Disease duration, months | 61 (19,120) | 84 (31,137) | 0.020 |
| Positive RF, n (%) | 694 (69.1) | 100 (80.0) | 0.012 |
| Positive ACPA, n (%) | 732 (72.8) | 87 (69.6) | 0.445 |
| ESR, mm/h | 36 (19,68) | 50 (32,91) | <0.001 |
| CRP, mg/L | 5.38 (3.16,25.75) | 16.40 (5.50,50.60) | <0.001 |
| CDAI | 13 (5,25) | 16 (7,30) | 0.026 |
| Active RA, n (%) | 819 (81.5) | 111 (88.8) | 0.044 |
| HAQ-DI | 0.38 (0.00,1.00) | 1.00 (0.25,1.50) | <0.001 |
| mTSS | 11 (3,33) | 13 (5,48) | 0.205 |
| Previous medications |  |  |  |
| Treatment naïve^△^, n (%) | 229 (22.8) | 30 (24.0) | 0.761 |
| Glucocorticoid, n (%) | 488 (48.6) | 61 (48.8) | 0.959 |
| Methotrexate, n (%) | 572 (56.9) | 70 (56.0) | 0.846 |
| Leflunomide, n (%) | 431 (42.9) | 61 (48.8) | 0.208 |
| Hydroxychloroquine, n (%) | 217 (21.6) | 10 (8.0) | <0.001 |
| Sulfasalazine, n (%) | 56 (5.6) | 3 (2.4) | 0.197 |
| Cyclosporine A, n (%) | 36 (3.6) | 4 (3.2) | 1.000 |
| TNF inhibitors, n (%) | 26 (2.6) | 3 (2.4) | 1.000 |
| Tocilizumab, n (%) | 30 (3.0) | 4 (3.2) | 0.784 |
| Janus kinase inhibitors, n (%) | 32 (3.2) | 4 (3.2) | 1.000 |
| Statin, n (%) | 62 (6.2) | 33 (26.4) | <0.001 |
| Aspirin, n (%) | 16 (1.6) | 17 (13.6) | <0.001 |
| CVD risk factors |  |  |  |
| Active smoking, n (%) | 150 (14.9) | 40 (32.0) | <0.001 |
| BMI, kg/m^2^ | 21.8±3.0 | 22.2±3.3 | 0.286 |
| Hypertension, n (%) | 289 (28.8) | 76 (60.8) | <0.001 |
| T2DM, n (%) | 120 (11.9) | 38 (30.4) | <0.001 |
| TC, mmol/L | 5.03±1.14 | 5.14±1.57 | 0.439 |
| TG, mmol/L | 1.17±0.79 | 1.33±0.78 | 0.035 |
| LDL-C, mmol/L | 3.15±0.81 | 3.21±1.13 | 0.565 |
| HDL-C, mmol/L | 1.38±0.38 | 1.32±0.44 | 0.106 |
| CKD, n (%) | 19 (1.9) | 14 (11.2) | <0.001 |
| Serum albumin, g/L | 34.7±5.9 | 32.5±6.1 | <0.001 |
| SUA, mg/dL | 4.83±1.61 | 5.57±2.31 | 0.001 |
| Hypouricemia, n (%) | 95 (9.5) | 25 (20.0) | <0.001 |
| Normouricemia, n (%) | 812 (80.8) | 62 (49.6) |  |
| Hyperuricemia, n (%) | 98 (9.8) | 38 (30.4) |  |

Abbreviations: RF, rheumatoid factor; ACPA, anti-cyclic citrullinated peptide antibody; ESR, erythrocyte sedimentation rate; CRP, C reactive protein; CDAI, clinical disease activity index; HAQ-DI, health assessment questionnaire disability index; mTSS, modified total Sharp score; TNF, Tumor necrosis factor; BMI, body mass index; T2DM, type 2 diabetes mellitus; TC, total cholesterol; TG, triglyceride; HDL-C, high-density lipoprotein cholesterol; LDL-C, low-density lipoprotein cholesterol; CKD, chronic kidney disease; SUA, serum uric acid.

**Additionl file 1: Table S2 Logistic regression analysis of the associations between SUA levels and CVD in RA patients**

| Characteristics | Univariate | |  | Multivariate | |
| --- | --- | --- | --- | --- | --- |
|  | OR (95% CI) | *P* |  | AOR (95% CI) ^*^ | *P* |
| **SUA levels^&^** |  |  |  |  |  |
| Hypouricemia | 3.645 (1.939,6.852) | <0.001 |  | 3.616 (1.732,7.552) | 0.001 |
| Normouricemia | Ref |  |  | Ref |  |
| Hyperuricemia | 3.591 (2.391,5.393) | <0.001 |  | 2.717 (1.714,4.306) | <0.001 |
| **SUA levels^#^** |  |  |  |  |  |
| SUA≤3.40 mg/dL | 2.910 (1.703,4.973) | <0.001 |  | 3.717 (1.994,6.928) | <0.001 |
| 3.40<SUA<5.20 mg/dL | Ref |  |  | Ref |  |
| SUA≥5.20 mg/dL | 2.940 (1.867,4.628) | <0.001 |  | 2.259 (1.339,3.810) | 0.002 |
| **SUA levels^$^** |  |  |  |  |  |
| SUA≤3.70 mg/dL | 3.048 (1.544,6.017) | 0.001 |  | 3.547 (1.693,7.428) | 0.001 |
| 3.70<SUA≤4.61mg/dL | Ref |  |  | Ref |  |
| 4.61<SUA<5.96 mg/dL | 2.006 (0.978,4.115) | 0.058 |  | 1.751 (0.800,3.834) | 0.161 |
| SUA≥5.96 mg/dL | 5.575 (2.916,10.659) | <0.001 |  | 3.775 (1.875,7.601) | <0.001 |

Abbreviations: SUA, serum uric acid; OR, odds ratio; AOR, adjusted OR; CI, confidence interval.

* Adjusted by age, gender (male or female), active smoking (yes or no), BMI, hypertension (yes or no), T2DM (yes or no), TC, TG, LDL-C, HDL-C, serum albumin and CKD (yes or no), RA disease duration, RF positivity (yes or no), ACPA positivity (yes or no), ESR, CRP, CDAI, HAQ-DI, mTSS, and previous treatment.

& Hypouricemia was defined as SUA level ≤ 3.0 mg/dL for male and ≤ 2.5 mg/dL for female, while hyperuricemia was defined as SUA ≥ 7.0 mg/dL for males and ≥ 6.0 mg/dL for females.

# Categorized according to the result of restricted cubic spline regression.

$ Categorized according to the 25^th^, 50^th^ and 75^th^ percentiles of SUA levels.
